# In vitro construction of liver organoids with biomimetic lobule structure by a multicellular 3D bioprinting strategy

**DOI:** 10.1111/cpr.13465

**Published:** 2023-05-17

**Authors:** Honglei Jian, Xin Li, Qianqian Dong, Shaonan Tian, Shuo Bai

**Affiliations:** ^1^ State Key Laboratory of Biochemical Engineering, Institute of Process Engineering Chinese Academy of Sciences Beijing China; ^2^ University of Chinese Academy of Sciences Beijing China; ^3^ Modular Platform Public Instrument Center Institute of Process Engineering, Chinese Academy of Sciences Beijing China

## Abstract

Liver disease is one of the serious threats to human life and health. Three‐dimensional (3D) liver models, which simulate the structure and function of natural liver tissue in vitro, have become a common demand in medical, scientific and pharmaceutical fields nowadays. However, the complex cellular composition and multi‐scale spatial arrangement of liver tissue make it extremely challenging to construct liver models in vitro. According to HepaRG preference and printing strategy, the formulation of bioink system with opposite charge is optimized. The sodium alginate‐based bioink 1 and dipeptide‐based bioink 2 are used to ensure structural integrity and provide flexible designability, respectively. The HepaRG/HUVECs/LX‐2‐laden liver organoids with biomimetic lobule structure are fabricated by a multicellular 3D droplet‐based bioprinting strategy, to mimic the cell heterogeneity, spatial structure and extracellular matrix (ECM) features. The liver organoids can maintain structural integrity and multicellular distribution within the printed lobule‐like structure after 7 days of culture. Compared with the 2D monolayer culture, the constructed 3D organoids show high cell viability, ALB secretion and urea synthesis levels. This study provides a droplet‐based and layer‐by‐layer 3D bioprinting strategy for in vitro construction of liver organoids with biomimetic lobule structure, giving meaningful insights in the fields of new drugs, disease modelling, and tissue regeneration.

## Graphical abstract

This study aims to in‐vitro construct of liver organoids with biomimetic lobule structure by a multicellular droplet‐based 3D bioprinting strategy consisting of hepatic parenchymal HepaRG cells, stellate cells and human umbilical vein endothelial cells, and deposition of multi‐bioinks in a layer‐by‐layer way forming a self‐standing construction with a hexagonal structure.

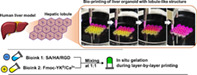

## 1 INTRODUCTION

The liver, as the largest metabolic organ of human body, is responsible for performing a variety of complex functions such as biosynthesis, secretion, storage, metabolism and detoxification. Unfortunately, orthotopic liver transplantation (OLT) is still the only effective treatment for end‐stage liver disease. However, the problems of donor organ shortage, matching type and immune rejection greatly limit its wide application.^1^, ^2^ Over the past few decades, liver modelling using human cells in petri dishes has contributed greatly to a variety of important research related to liver disease treatment and liver regeneration.^3^, ^4^, ^5^

Organoid models have gradually matured and become prominent in recent years.^6^, ^7^, ^8^, ^9^ Compared with traditional monolayer culture and animal models, organoids are able to partially reproduce key physiological features of the native human organs, including cell heterogeneity, spatial structure and microenvironment, and provide important cell–cell and extracellular matrix (ECM) interactions.^10^, ^11^ These can promote hepatocyte proliferation, differentiation, expression of specific genes and proteins, and response to exogenous stimuli.^12^

3D bioprinting is a promising technique that enables spatial patterning of multiple cells and matrix materials in a preset manner, offering unprecedented potential for creating organoids that mimic the microscopic structure of native organs.^5^, ^13^, ^14^ Bioprinting of hepatic and endothelial cells to produce structured spheroids has showed better performance including long‐term culture, cell viability and high MRP2, albumin (ALB), and CD31 expression levels when compared to the non‐structured spheroids.^15^ A hepatic construct with hepatocyte progenitor cells having a hexagonal structure has been developed by a two‐step bioprinting strategy, which showed significantly improved hepatocyte phenotype and function.^16^ An array of liver lobules prepared using an extrusion‐based bioprinting method demonstrated that the multicellular types with spatial patterns in bioinks play a role in cell organization and function.^17^ In addition, a liver model constructed by extrusion bioprinting showed clinically relevant dose‐dependent hepatotoxicity.^18^ Moreover, in a bioprinting study, the multicellular composition of hepatic parenchymal, stellate and endothelial cells was shown to be necessary to construct a liver fibrogenesis model.^19^

However, due to the complex cellular composition of liver tissue and the multi‐scale spatial arrangement between cell and ECM, the challenges for 3D bioprinting of liver models are mainly limited by the performance of bioink materials. The characteristics of bioink materials not only need to meet the printability requirements, but also need to meet the bionic needs of different cell types for specific ECMs. So far, few engineered liver models have been able to simultaneously mimic multicellular composition, spatial patterns, and ECM characteristics of natural livers.

Hence, this study aims to in vitro construction of liver organoids with biomimetic lobule structure by a multicellular droplet‐based 3D bioprinting strategy. According to the literature,^19^ the multicellular composition of the 3D liver model consists of hepatic parenchymal HepaRG cells, stellate cells (LX‐2) and human umbilical vein endothelial cells (HUVECs). The selected multi‐material bioinks, including sodium alginate (SA), hyaluronic acid (HA) and dipeptide (with preferred sequence by HepaRG cells), are deposited in a layer‐by‐layer way eventually to form a self‐standing construct with defined structure. The multicellular 3D printing and bioink material design strategies developed in this study have great potential in the development of 3D organoids that mimic the native microenvironment of organs.

## 2 MATERIALS AND METHODS

### 2.1 Cell culture

The used cells in this study, including HepaRG, LX‐2, HUVECs, green‐fluorescent HepaRG (HepaRG‐GFP), bule‐fluorescent LX‐2 (LX‐2‐BFP), and red‐fluorescent HUVECs (HUVECs‐RFP), were cultured in DMEM containing 4.5 g/L d‐glucose, supplemented with 10% (v/v) FBS (Gibco) and 1% (v/v) penicillin–streptomycin at 37°C with 5% CO_2_ in an incubator. In the experiment using 2D culture as a control, the 2D monolayer culture group maintained the same HepaRG cell number as the 3D model. During cell culture, half of culture medium was replaced by the fresh one every 2 days.

### 2.2 Preparation of cell‐laden bioinks

Formulation of each cell‐laden bioink used in 3D bioprinting was listed in Table 1. Bioink 1 was composed of sodium alginate (SA, 15–25 cP, CAS: 9005‐38‐3), hyaluronic acid (HA, M_W_ = 4 × 10^5^ Da, CAS: 9004‐61‐9) and Arg‐Gly‐Asp (RGD) sequence peptide, and bioink 2 was consisted of Fmoc‐Tyr‐Lys (Fmoc‐YK^*d*^) and calcium chloride. The electrostatic interaction between negatively charged bioink 1 and positively charged bioink 2 ensures in‐situ gelation to preserve the fabricated structure. For the cell‐laden bioinks, HepaRG cells were encapsulated in bioink 1 at a final density of 7.5 × 10^6^ cells/mL. LX‐2 and HUVECs were respectively embedded in bioink 2 to achieve a final density of 1 × 10^7^ and 2.5 × 10^6^ cells/mL, respectively. The used ratio of HepaRG/HUVECs/LX‐2 in constructed hydrogel scaffold was maintained approximately 10:8:3 during the printing process.

**TABLE 1:** Formulation of the cell‐laden bioinks in 3D bioprinting of liver organoids.

bioinks	Cell density (cells/mL)	SA(w/v%)	HA (w/v%)	RGD (mg/mL)	Fmoc‐YK (mM)	Ca^2+^ (mM)
HepaRG‐laden bioink 1	7.5 × 10^6^	0.5	0.125	0.5	–	–
LX‐2‐laden bioink 2	1 × 10^7^	–	–	–	10	50
HUVECs‐laden bioink 2	2.5 × 10^6^	–	–	–	10	50

### 2.3 Characterizations of the prepared bioinks

The prepared bioinks were characterized by zeta potential measurements, atomic force microscopy (AFM) imaging and rheological analysis. The bioinks were diluted for 20‐fold by Milli‐Q water to zeta potential measurements on a Zetasizer Nano‐ZS (Malvern) at 25°C. For AFM imaging, 5 μL of bioink samples were deposited on mica sheets. After air‐dried, samples were observed using FASTSCANBIO AFM (Bruker) in a tapping mode, and the obtained images were processed using the nano‐scope analysis 1.9 software (Bruker). The rheological analysis including amplitude and frequency sweeps was performed on a MCR 302 rheometer (Anton Paar) equipped with a 25 mm diameter parallel plate at a 0.5 mm gap. The test temperature was maintained at 25 ± 3°C. The amplitude sweep was carried out in the strain range of 0.01%–100% with a fixed frequency of 1 Hz. The frequency sweep was performed in the range of 0.1–100 Hz at a constant shear strain of 1%.

### 2.4 Model designing and 3D bioprinting

The liver model with biomimetic lobule structure consisted of 12 hexagons, three cell types, and two formulated bioinks. A layer‐by‐layer bioprinting strategy was conducted by a droplet‐based Digilab CELLJET™ 3D bioprinter (Thermo Fisher Scientific) equipped with a 190 μm inner‐diameter nozzle. Prior to printing, the prepared bioinks were sterilized for 30 min under UV light and then loaded into the dispensing system by the syringe pump through the tip orifice. The dispense positions were programmed as coordinates by AxSys™ software, and the dispense volume for each drop was optimized as 50 nL. The spacing distance between droplets is 375 μm. The diameter of each droplet formed in the constructed hydrogel scaffold is approximately 294 ± 11 μm.

### 2.5 Swelling and degradation of hydrogel scaffold

The swelling and degradation of hydrogel scaffolds were studied by gravimetric and rheological methods. The initial weight of the fabricated hydrogel scaffold was marked as *W*_i_. During test, printed hydrogel scaffolds were immersed in PBS solution and maintained at 37°C. At different time points (Day 1, 5 and 10), the liquid on hydrogel surface was gently removed by filter paper, and the wet weight of the hydrogel scaffold was measured as *W*_t_. The swelling and degradation of hydrogel scaffold were evaluated by wet weight gain% = (*W*_t_ − *W*_i_)/*W*_i_ × 100%. At each time point (Day 0, 1, 5 and 10), the elastic modulus (G′) of hydrogel scaffold was determined by the rheological method described above.

### 2.6 Cell viability and proliferation

At days 1 and 7 of the culture period, cell viability of the constructed 2D or 3D models was evaluated by a live‐dead staining of green‐fluorescent Calcein AM and red‐fluorescent PI for 40 min at 37°C. Images of the stained samples were captured by LSM 710 confocal laser scanning microscope (Zeiss) and further processed by ZEN software. Cell proliferation was determined by Cell Counting Kit‐8 (CCK‐8; DOJINDO) on days 1, 4 and 7 during culture. Briefly, 10% (v/v) CCK‐8 solution was added to a petri dish and incubated at 37°C and 5% CO_2_ for 4 h. The absorbance at 450 nm of the sample was immediately recorded by a microplate reader (Thermo Scientific).

### 2.7 Immunofluorescence staining

For immunofluorescence staining, the fabricated liver organoids matured for 7 days were fixed in 4% paraformaldehyde solution for 30 min, permeabilized in 0.5% (v/v) Triton X‐100 solution for 20 min, and subjected to blocking in 2% (v/v) BSA solution. The hepatic cells of the constructed liver models were immunolabelled by ALB. The cell‐laden samples were incubated with the primary antibodies of rabbit anti‐albumin (ab207327, abcam, 1:400 dilution) overnight at 4°C. Afterwards, the samples were treated with the green‐fluorescent goat anti‐rabbit (ab150117, abcam) secondary antibody for 3 h. The cell nuclei were counterstained with DAPI. The z‐stacking images the stained samples were captured by LSM 710 CLSM and processed using ZEN software.

### 2.8 In vitro analysis of hepatic functions

For hepatic function measurements, the ALB secretion and urea synthesis were quantified using the corresponding assay kits following the protocol from the manufacturer. At days 1, 4, and 7 during culture, the supernatant was collected and stored at −80°C. The insoluble matter of the sample was centrifugally removed prior to testing. ALB and urea secretions in the supernatant were determined using the ELISA kit (ab179887, Abcam) and urea assay kit (ab83362, Abcam), respectively. ALB and urea contents were assayed by absorbance at 450 nm and 462 nm on a microplate reader (Thermo Scientific), respectively.

### 2.9 Statistical analysis

All tests were repeated three times in parallel, and all data in this study were presented as the mean value ± standard deviation. The statistical analysis was performed using one‐way ANOVA test to determine the significant levels at **p < 0.01 and *p < 0.05.

## 3 RESULTS

### 3.1 Bioink formulation and characterizations

In the liver organoid construction, two bioinks were required to load HepaRG and LX‐2/HUVECs cells, respectively. The bioink 1, composed of SA/HA/RGD, was used to encapsulate HepaRG cells. The dipeptide material was used as bioink 2 to respectively load LX‐2/HUVECs cells. The design inspiration for this bioink system is as follows: in view of the complex structure to be constructed and the regionalized spatial distribution of the three types of cells, it seems necessary to use polymeric materials in combination with supramolecular materials. As shown in Figure S1, we chose the classic crosslinking between SA and calcium as the basis, and the brittleness and viscoelasticity (crosslinking shrinkage) of the gel network is improved by adding HA. Supramolecular hydrogels are then added to the bioink system, given that the interaction between cells and matrix in vivo is dynamic. In the final bioink system, the excellent mechanical properties of the natural polysaccharide bioinks (SA and HA) can ensure the structural integrity of the construct during the subsequent culture period. Short peptide (Fmoc‐YK) supramolecular hydrogel can be used to simulate the dynamic network characteristics of cell‐matrix interactions, providing good cytocompatibility and degradation performance.

**FIGURE 1:**
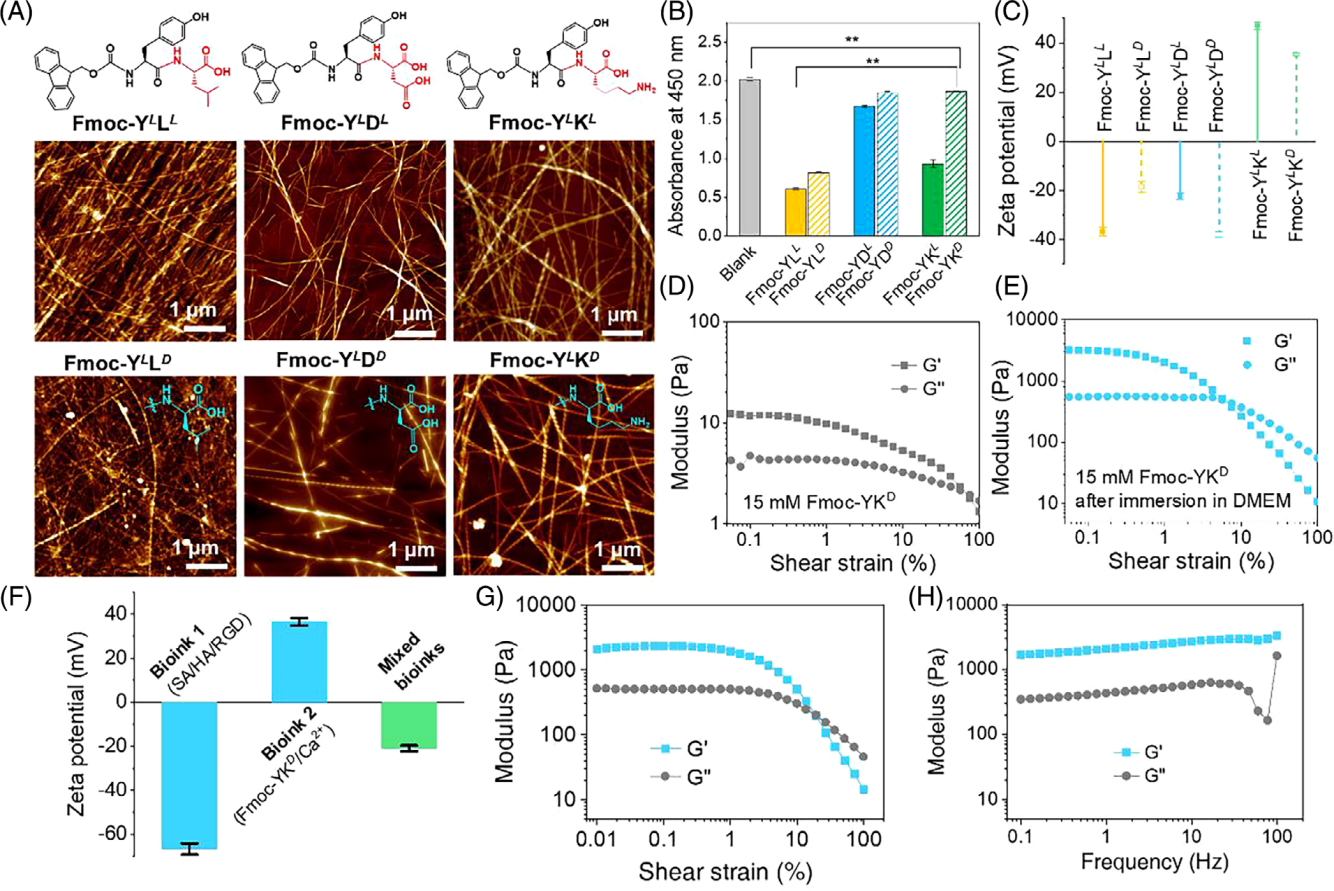
Studies on the bioink formulation, determination of the preference of HepaRG cells on the dipeptide bioinks with different sequences. (A) Molecular structure and AFM images of the Fmoc‐dipeptides. (B) Cytocompatibility of dipeptide materials on HepaRG cells after 24 h of incubation. The asterisks in figure indicate the level of significant difference (*p < 0.05, **p < 0.01). (C) Zeta potentials of Fmoc‐dipeptide solutions at a concentration of 0.5 mM. (D) Mechanical strength of 15 mM Fmoc‐YK^*d*^ hydrogel and (E) the mechanical strength enhancement after immersion in DMEM. (F) Zeta potentials of bioinks 1, 2 and the mixture of bioink 1/bioink 2 at a volume ratio of 1:1. Bioink 1 was composed of 0.5% sodium alginate (SA), 0.125% hyaluronic acid (HA) and 0.5 mg/mL Arg‐Gly‐Asp (RGD). Bioink 2 was consisted of 10 mM Fmoc‐YK^*d*^ and 50 mM calcium chloride. The bioink samples were diluted for 20‐fold prior to the zeta potential determination. (G) Amplitude and (H) frequency sweeps on the hydrogel prepared by mixing bioinks 1 and 2 at a volume ratio of 1:1.

As shown in Figure 1, three dipeptide sequences (including Fmoc‐YL, YD and YK) with different chirality were designed to verify the preference of HepaRG cells on them. The molecular structure and AFM images of the Fmoc‐dipeptides were shown in Figure 1A. All the peptide sequences were assembled into nanofiber networks with different structures. The results of cell proliferation (Figure 1B) and live‐dead staining (Figure S2) illustrated that HepaRG cells showed a preference for the three dipeptides of Fmoc‐YD^*l*^, YD^*d*^ and YK^*d*^. In this study, we hope that this ink material can be charge neutralized with negatively charged bioink 1 to form a gel (Figure 1C). In particular, Fmoc‐YK^*d*^ (15 mM) can self‐assemble into hydrogel in aqueous sodium hydroxide solution with a pH value of about 4.5, and the mechanical properties of the gel can be improved after immersion in the cell culture medium of DMEM (Figure 1D,E). This is a very useful property for Fmoc‐YK^*d*^ in cell culture applications. Therefore, the formulation of bioink 2 was determined to be 10 mM positively charged Fmoc‐YK^*d*^ mixed with 50 mM calcium chloride.

Zeta potentials of bioinks 1 and 2 were − 66.6 ± 2.6 and 36.4 ± 1.7 mV, respectively (Figure 1F). The electrostatic interaction between the two bioinks changed the zeta potential value to −21.0 ± 1.3 mV. The mechanical properties, including storage modulus (G′) and loss modulus (G″), of the prepared bioinks were determined. Individual bioinks 1 and 2 were both liquids with fluidity, and their rheological curves were shown in Figure S3. Mixing bioinks 1 and 2 at a volume ratio of 1:1 resulted in a sol–gel transition, with G′ showing a stable value of approximately 2.3 kPa in the platform region (Figure 1G,H), which indicated the electrostatic interaction between the negatively charged bioink 1 and positively charged bioink 2 after mixing.

### 3.2 Generation of liver organoids

**FIGURE 2:**
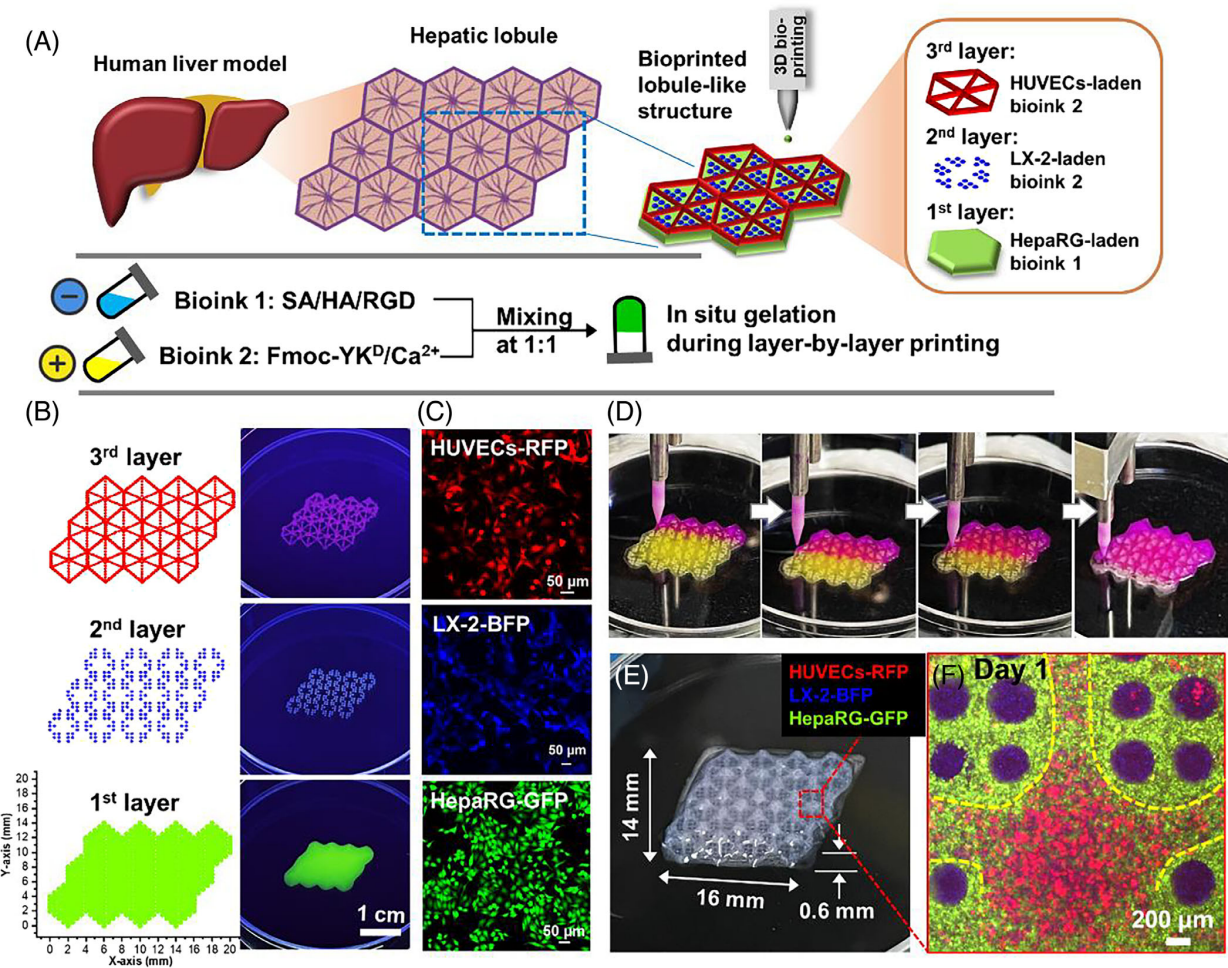
(A) Schematic diagram of in vitro construction of liver organoids with biomimetic lobular structure by a multicellular 3D bioprinting strategy. (B) Programming of 3D droplet‐based bioprinting for the in vitro liver model fabrication. (C) Multicellular composition of the constructed liver organoid: HUVECs‐RFP, LX‐2‐BFP, and HepaRG‐GFP. (D) Photos of the printing process. (E) Optical image of 3D constructed liver organoid with hydrogel scaffold. (F) Confocal image of the hexagonal central region in the constructed lobule‐like structure after 1 day of culture.

A layer‐by‐layer bioprinting strategy was conducted by a droplet‐based 3D bioprinter equipped with a 190 μm inner‐diameter nozzle. The schematic diagram of in vitro construction of liver organoids with biomimetic liver lobular structure was shown in Figure 2A. The dispense positions of the three layers were programmed as coordinates respectively (Figure 2B), and the dispense volume for each drop was optimized as 50 nL. The swelling and degradation of hydrogel scaffolds were studied by gravimetric and rheological methods. As can be seen from Figure S5, the weight of the hydrogel scaffold increased by 94.6 ± 6.5% after 1 day of PBS immersion, indicating that the hydrogel network was mainly in the stage of water absorption and swelling. On Day 5, the printed scaffold showed significant decrease in both the weight gain and mechanical strength, indicating that the hydrogel swelling and erosion occurred simultaneously. On Day 10, the weight of the scaffold was lower than the initial value, which demonstrated that erosion of the hydrogel network became the dominant mechanism of degradation.

**FIGURE 3:**
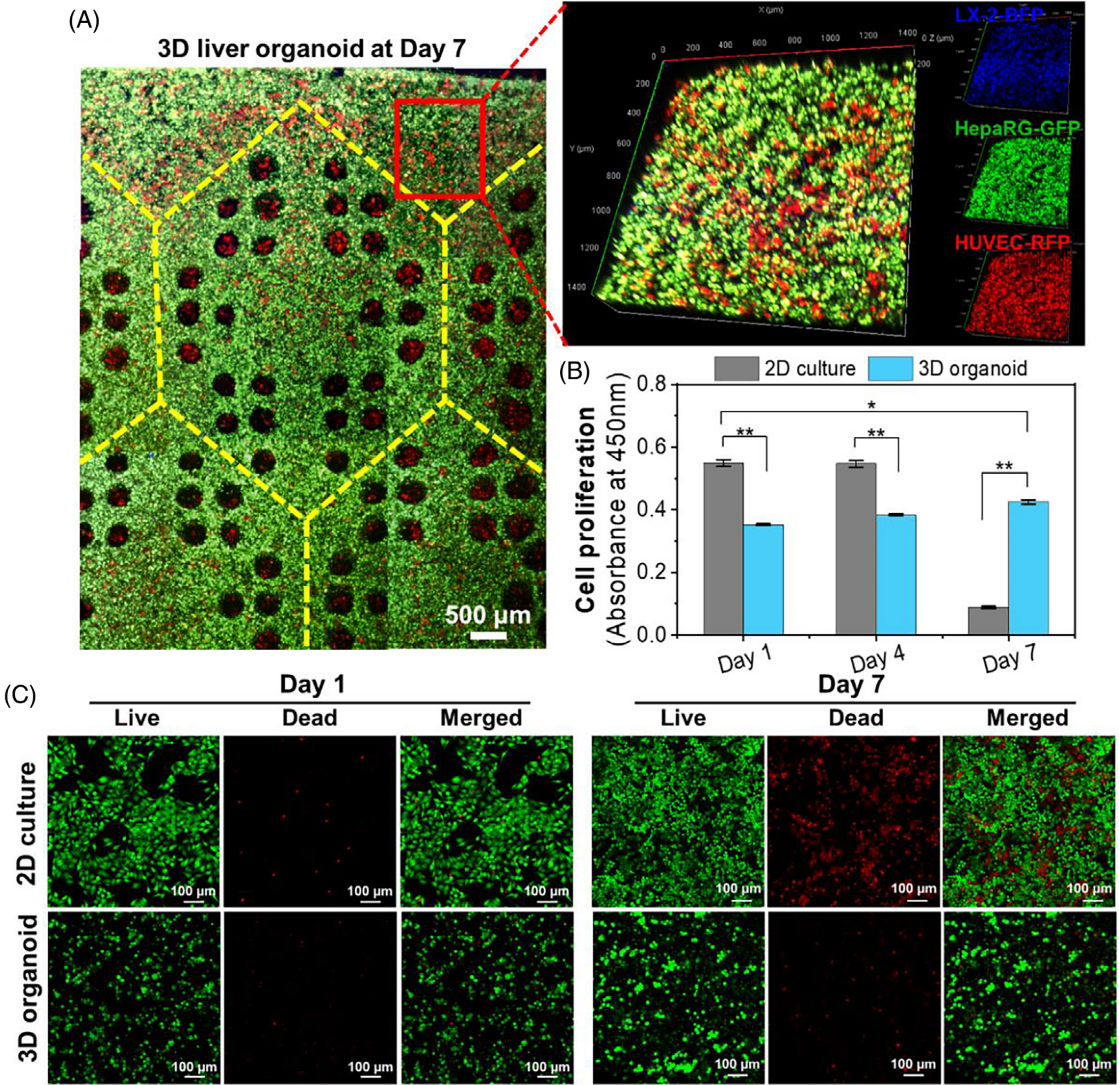
(A) Confocal images of the constructed liver organoids by multicellular 3D printing with HepaRG, LX‐2 and HUVECs cells, after 7 days of culture. (B) Cell proliferation determined by the absorbance at 450 nm. The asterisks in figure indicate the level of significant difference (*p < 0.05, **p < 0.01). (C) Live‐dead stain of 2D culture and the constructed liver organoids on days 1 and 7 during culture.

Fluorescent transfected cells, including HepaRG‐GFP, LX‐2‐BFP and HUVECs‐RFP cells, were used to illustrate the multicellular composition (Figures 2C), the lobule‐like structure and cell distributions in the printed model at days 1 and 7 (Figures 2F and 3A). During the construction of liver organoids, the layer‐by‐layer printing process was shown in Figure 2D. As can be seen from Figures 2E and S4, the dimensions of the constructed model were approximately 16 mm (length) × 14 mm (width) × 0.6 mm (thickness). The shape fidelity of the fabricated construct was assessed by the ratio of the actual dimension to the theoretical dimension of the digital model used for fabrication.^20^, ^21^ The shape fidelity of the printed model in the x‐axis and y‐axis directions was 104.6 ± 1.1% and 104.5 ± 1.2%, respectively. The confocal image of the hexagonal central region in the constructed lobule‐like structure after 1 day of culture was shown in Figure 2F. It can be seen that three fluorescent cells were spatially well‐organized in the printed structure.

### 3.3 Cell distribution, viability and proliferation

Figure 3A showed the cellular organization and distribution of the liver organoids with biomimetic lobule structure, after 7 days of culture. During culture, the three cells spontaneously organized together. Cell proliferation (Figure 3B) assayed by absorbance at 450 nm was in accordance with the live‐dead stain results (Figure 3C). Since 2D culture was used as a control for 3D models, the initial cell density was relatively high compared to conventional 2D monolayer culture. As demonstrated in Figure 3B, the number of cells in 2D culture remained dynamically stable during the first 4 days, but on Day 7, a large number of cells died due to contact inhibition (Figure 3B,C). Within 7 days, the cells embedded in the liver organoids showed a steady proliferation and maintained viability (Figure 3B,C).

### 3.4 ALB and urea secretions

**FIGURE 4:**
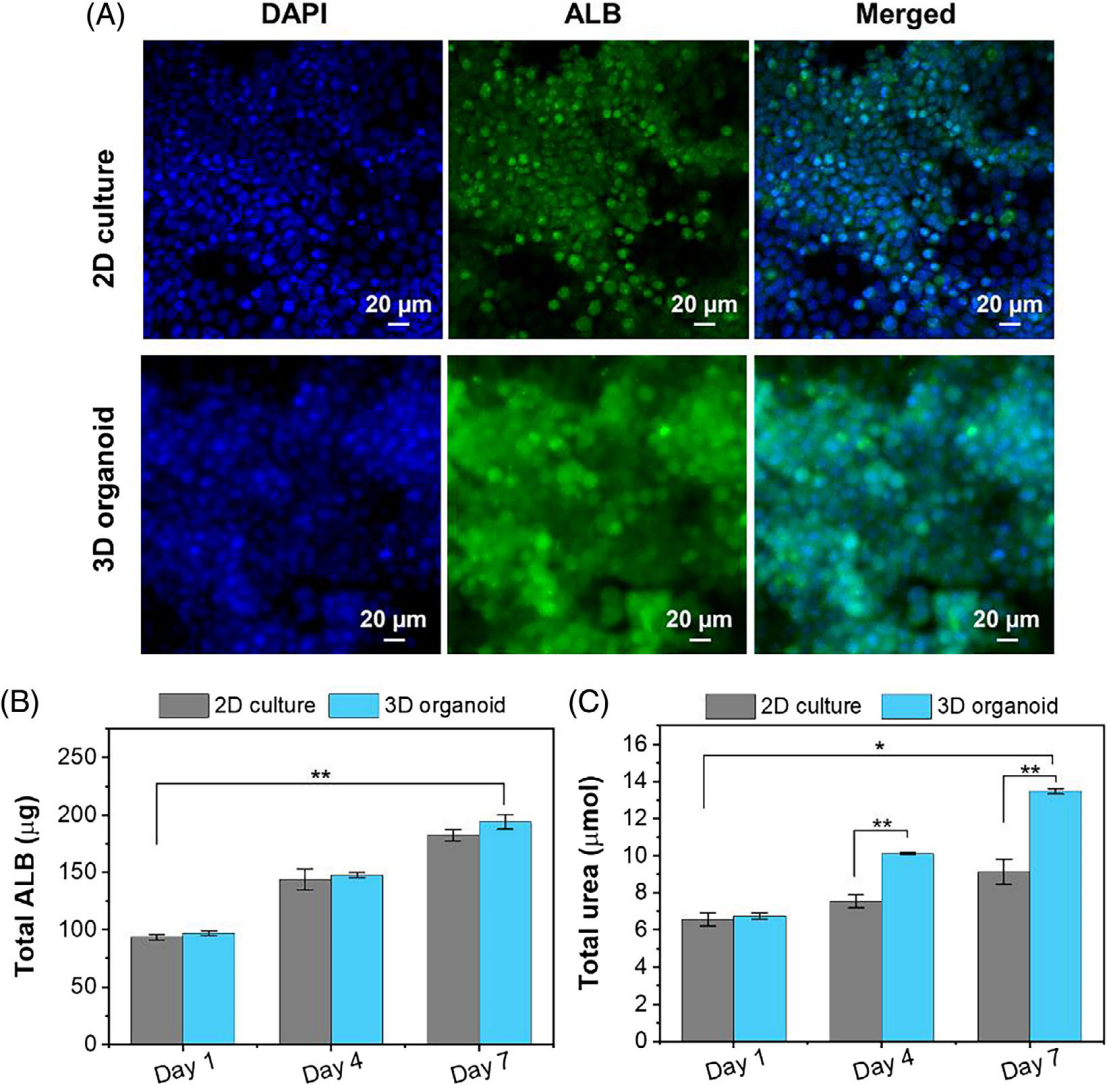
Hepatic function evaluations of 2D and 3D models. (A) Immunostaining of ALB (green) and nuclei (bule, DAPI) of cells on Day 7 during culture period. Determination of the total (B) ALB and (C) urea secretions in the supernatant at days 1, 4 and 7 during culture. The asterisks in figure indicate the level of significant difference (*p < 0.05, **p < 0.01).

The ALB secretion and urea synthesis of the HepaRG/HUVECs/LX‐2‐laden liver model were determined by immunofluorescence staining and quantitative assay kits. Figure 4A showed that the ALB secretion in 3D organoids was significantly higher than that in 2D culture. As can be seen from Figure 4B,C, ALB and urea secretions in both liver models were time‐dependent and increased significantly with the prolonged culture during 7 days. On Day 7, the total content of ALB secreted in the supernatant from 2D culture and 3D organoid models was 182.1 ± 6.3 and 194.0 ± 5.2 ng, respectively (Figure 4B). The total urea secretion in the supernatant of the 2D and 3D models was 9.1 ± 0.1 and 13.5 ± 0.1 μmol on Day 7, respectively (Figure 4C).

## 4 DISCUSSION

From a physiological point of view, hepatocytes are located in a complex but well‐organized microenvironment provided by the lobules, including various types of non‐parenchymal (such as endothelial, stellate and Kupper) cells, insoluble ECMs and soluble cytokines. All these factors are involved in regulating the phenotype and function of hepatocytes.^22^, ^23^, ^24^ ECMs distributed around cells not only provide mechanical support for tissues and organs, but also participate in a variety of cell signalling pathways.^7^, ^25^ Therefore, the mimicking of cell heterogeneity, ECM composition and structural features of native liver organ is essential for the functional expression of the in vitro constructed models.^26^, ^27^

Currently, bioink materials used to load complex types of cells in organoid modelling are still very limited in variety and performance.^28^ Many existing 3D liver models rely on acellular matrices (e.g., Matrigel) as ECM‐mimics that support cells for physical attachment, allow organoids to self‐organize and differentiate within them, and provide factors and hormones that influence gene and protein expression.^29^, ^30^ However, animal‐derived hydrogels have drawbacks, including batch‐to‐batch variability and species differences, which severely limit their further use in vivo. The other commonly used polymeric bioinks, such as collagen, GelMA, hyaluronic acid and alginate, are also limited by batch‐to‐batch variability and lack of stimulus responsiveness and performance regulation. After printing, they usually rely on UV light, heat or chemicals to conduct post‐printing crosslinking. To a certain extent, it is unfavourable to cell vitality and printing fidelity.^31^

Supramolecular hydrogels are formed through noncovalent interactions (such as hydrogen bonding, aromatic stacking, electrostatic interactions, etc.) between the building blocks. Short peptide‐based supramolecular hydrogels have the potential to become novel bioink materials.^20^, ^32^ They usually have good biocompatibility, ordered and reversible nanostructures, and stimulus responsiveness to pH, temperature, light, and so forth. In this study, dipeptide materials were used as bioinks based on the preferred sequence by HepaRG cells and the printing strategy.

The liver model with lobule‐like structure was conducted by a droplet‐based and layer‐by‐layer bioprinting strategy.^33^, ^34^ Two bioinks were required to load HepaRG and LX‐2/HUVECs cells, respectively. In the formulation of bioink 1, sodium alginate was used to provide cross‐linked network structure, and hyaluronic acid to provide network viscoelasticity. Specifically, RGD peptide was added in bioink 1 to improve cell adhesion. The excellent mechanical properties of the natural polysaccharide bioinks can ensure the structural integrity of the construct during the subsequent culture period. The bioink 2 was based on dipeptide self‐assembly hydrogels, which need to be mixed with bioink 1 to form a gel in situ. It should be noted that the bioink system used in this study involves three crosslinking actions: The primary is the crosslinking between calcium ions and alginate; the second is between positively charged Fmoc‐YK^*d*^ fibres and alginate; the third is between Fmoc‐YK^*d*^ and DMEM in cell culture process. At the beginning, cells in the 3D model may need to adapt to the constructed hydrogel environment, including the establishment of cell–cell and cell‐matrix interactions and ECM secretions. Within the following culture for 7 days, the embedded cells in 3D organoids maintained a steady proliferation, while a large number of cells died due to contact inhibition in 2D cell culture.

In summary, the liver organoids can maintain structural integrity and multicellular distribution within the printed lobule‐like structure after 7 days of culture. Compared with the 2D monolayer culture, the constructed 3D organoids show high cell viability, ALB secretion and urea synthesis levels. The multicellular 3D printing and bioink material design strategies developed in this study have great potential in the development of 3D organoids that mimic the native microenvironment of organs. The findings of this study have great significance in the field of tissue engineering for applications in high‐throughput drug screening, disease modelling, as well as liver regeneration and treatment, and give meaningful insight in the field of new drugs and precision medicine.

## AUTHOR CONTRIBUTIONS

**Honglei Jian and Shuo Bai:** Conception, methodology, data analysis, interpretation, and manuscript writing. **Xin Li, Qianqian Dong and Shaonan Tian:** Data acquisition, analysis and interpretation. **Shuo Bai:** manuscript editing, and final approval of manuscript.

## FUNDING INFORMATION

This work was supported by the Strategic Priority Research Program of the Chinese Academy of Sciences (No. XDA16020808), the Chinese Major Program for the National Key Research and Development Project (Grant No. 2020YFA0112603), and the National Natural Science Foundation of China (No. 22277121).

## CONFLICT OF INTEREST STATEMENT

The authors declare no conflict of interest.

## Supporting information


**Data S1:** Supporting Information

## Data Availability

The data used to support the findings of this study are available from the corresponding author upon request.
