# Supplementary material for: In vitro construction of liver organoids with biomimetic lobule structure by a multicellular 3D bioprinting strategy
Source: Cell Prolif. 2023 May 17;56(5):e13465. doi: 10.1111/cpr.13465 (PMC10212698; doi:10.1111/cpr.13465)
Supplement: Supplementary file 1 — Data S1: Supporting Information [file CPR-56-e13465-s001.docx]

**Supporting Information**

**In vitro construction of** **liver organoids with biomimetic lobule structure by a multicellular 3D bioprinting strategy**

Honglei Jian^1^ | Xin Li^1,2^ | Qianqian Dong^1^ | Shaonan Tian^3^ | Shuo Bai^1,2,^*

^1^State Key Laboratory of Biochemical Engineering, Institute of Process Engineering, Chinese Academy of Sciences, Beijing, China

^2^University of Chinese Academy of Sciences, Beijing, China

^3^Modular Platform Public Instrument Center, Institute of Process Engineering, Chinese Academy of Sciences, Beijing 100190, China

**Correspondence**

Prof. Shuo Bai, State Key Laboratory of Biochemical Engineering, Institute of Process Engineering, Chinese Academy of Sciences, Beijing 100190, China.

Email: [baishuo@ipe.ac.cn](mailto:baishuo@ipe.ac.cn)


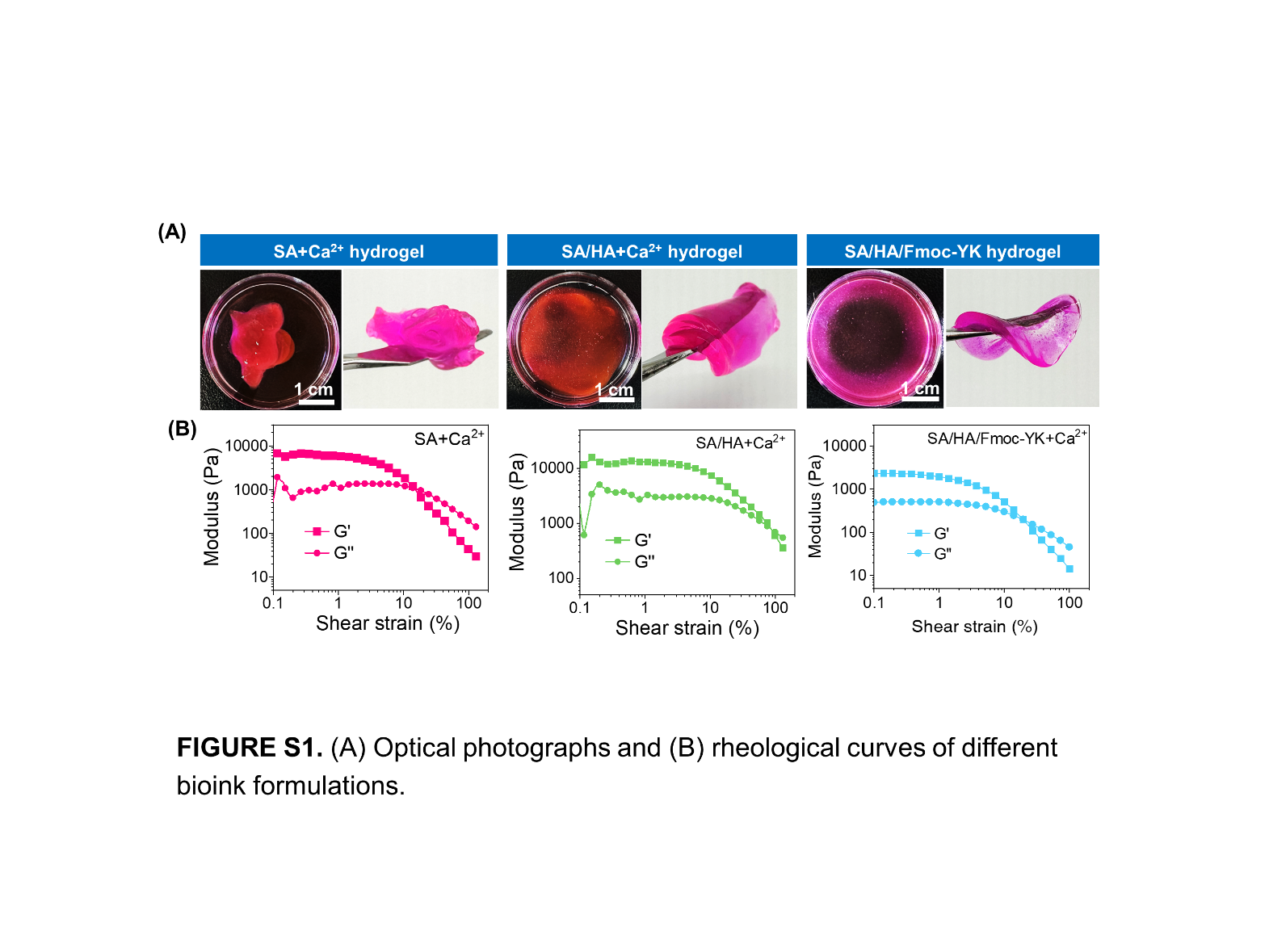


**FIGURE S1.** (A) Optical photographs and (B) rheological curves of different bioink formulations.


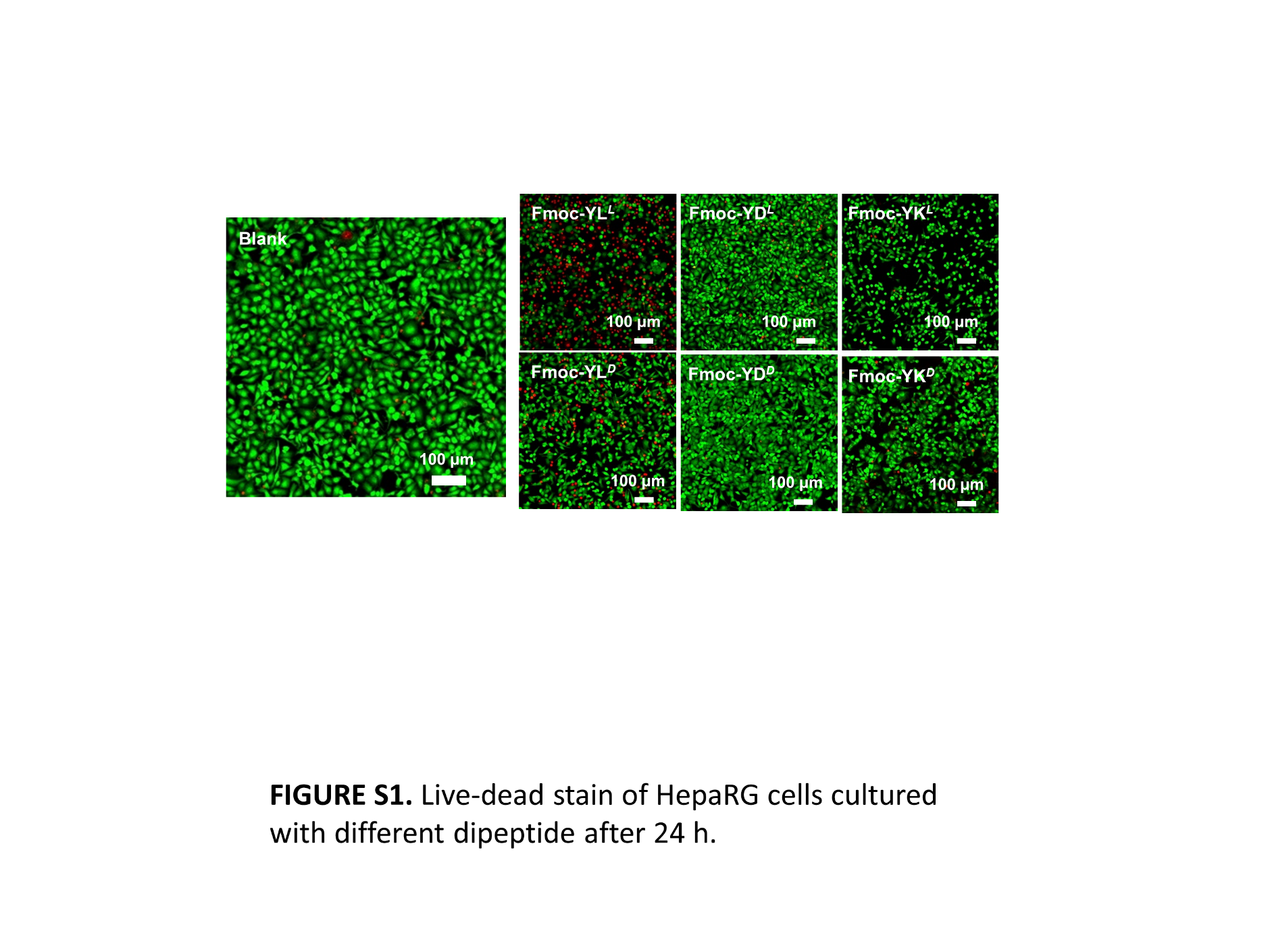


**FIGURE S2.** Live-dead stain of HepaRG cells cultured with different dipeptide after 24 h.


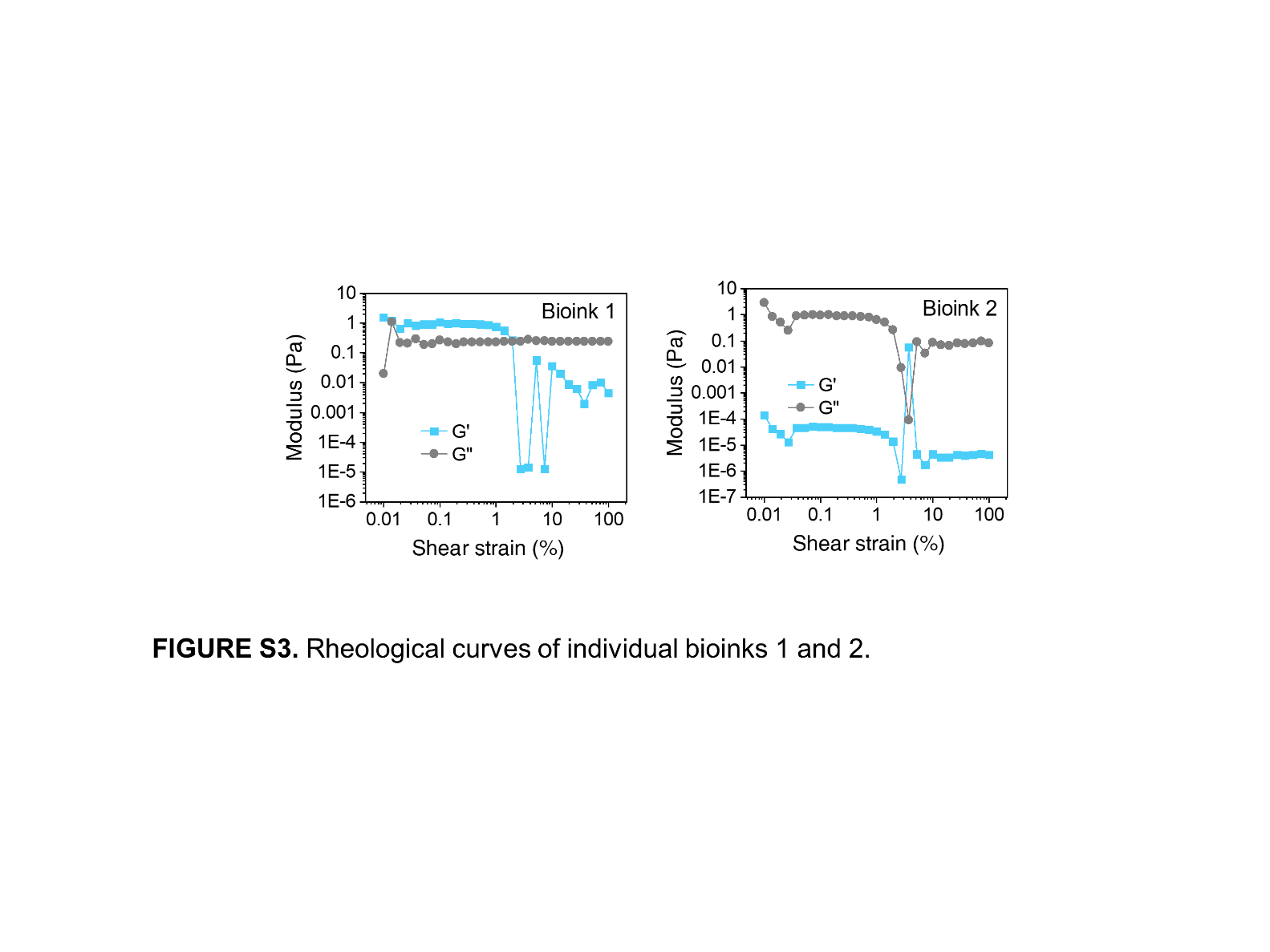


**FIGURE S3.** Rheological curves of individual bioinks 1 and 2.


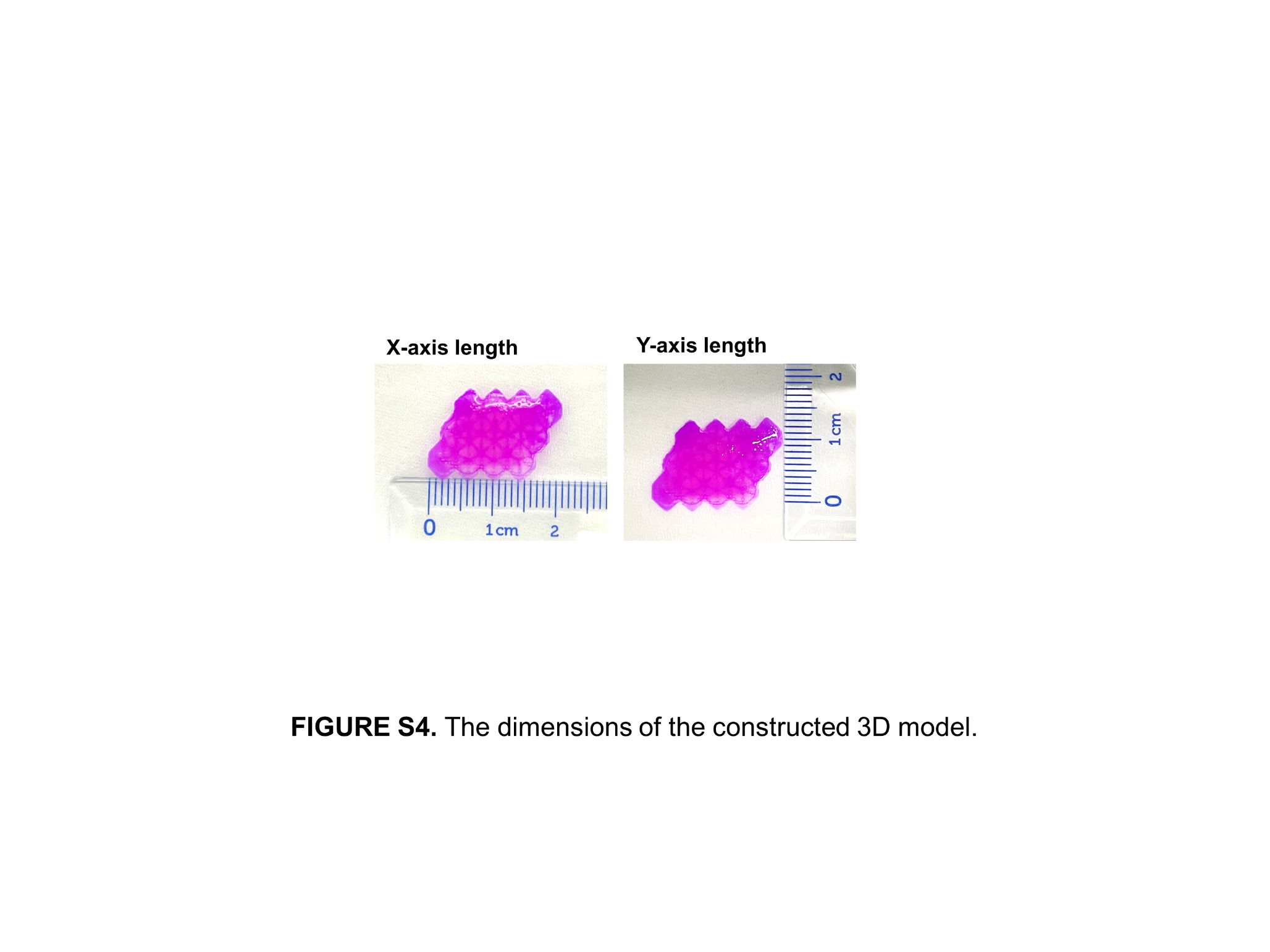


**FIGURE S4.** The dimensions of the constructed 3D model.


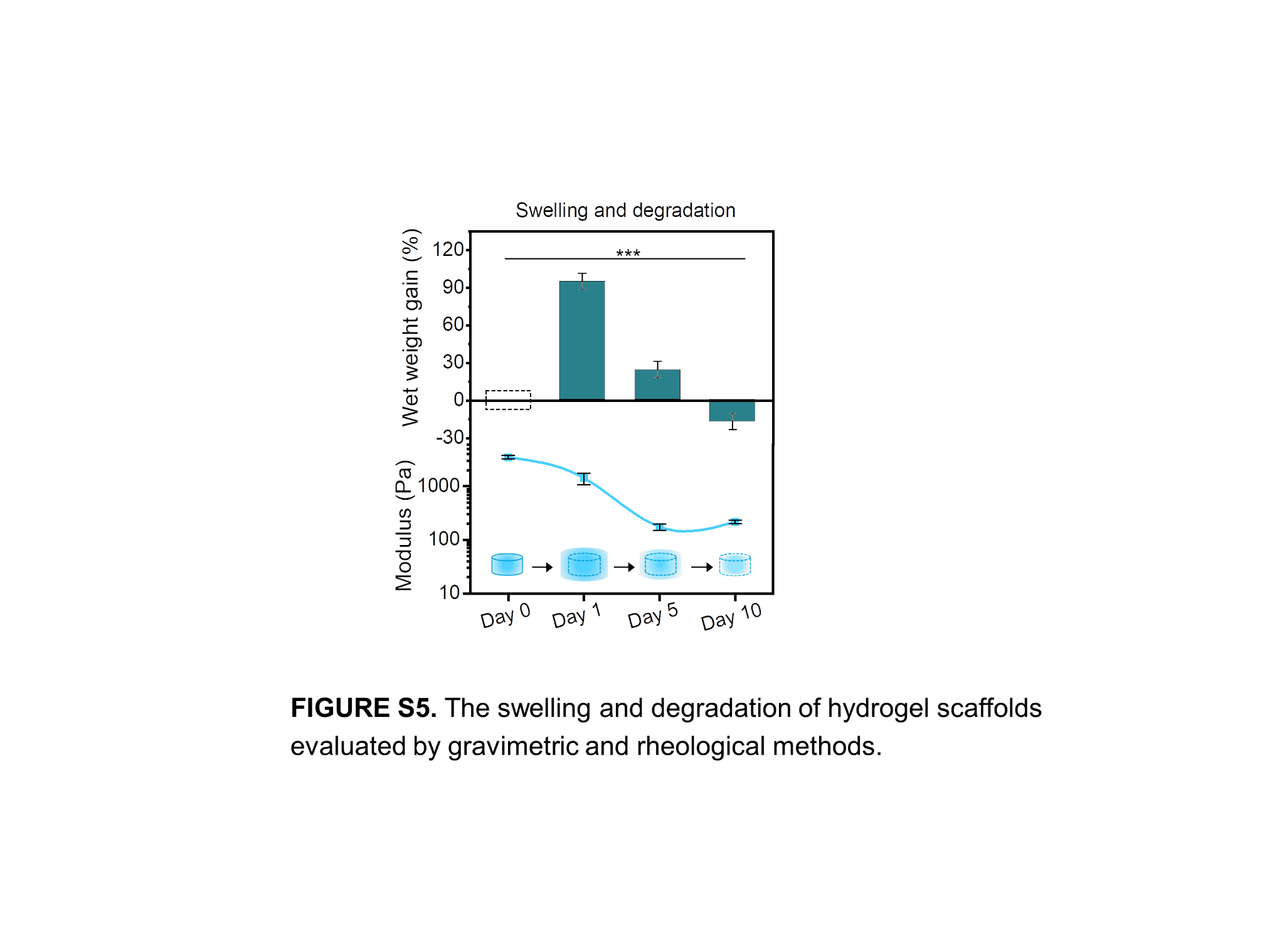


**FIGURE S5.** The swelling and degradation of hydrogel scaffolds evaluated by gravimetric and rheological methods.
